# The palmitoylation of the N-terminal extracellular Cys37 mediates the nuclear translocation of VPAC1 contributing to its anti-apoptotic activity

**DOI:** 10.18632/oncotarget.17449

**Published:** 2017-04-27

**Authors:** Rongjie Yu, Hongyu Liu, Xinhe Peng, Yue Cui, Suqin Song, Like Wang, Huahua Zhang, An Hong, Tianhong Zhou

**Affiliations:** ^1^ Institute of Biomedicine, School of Life Science and Technology, Jinan University, Guangzhou, Guangdong, China; ^2^ National Engineering Research Center of Genetic Medicine, Jinan University, Guangzhou, Guangdong, China; ^3^ Department of Medical Genetics, Guangdong Medical University, Dongguan, Guangdong, China; ^4^ Department of Bioengineering, School of Life Science and Technology, Jinan University, Guangzhou, Guangdong, China

**Keywords:** vasoactive intestinal peptide receptor 1 (VPAC1), cysteine (Cys), palmitoylation, nuclear translocation, anti-apoptotic activity

## Abstract

VPAC1 is class B G protein-coupled receptors (GPCR) shared by pituitary adenylate cyclase activating polypeptide (PACAP) and vasoactive intestinal peptide (VIP). The first cysteine (Cys37) in the N-terminal extracellular domain of mature VPAC1 is a free Cys not involved in the formation of conserved intramolecular disulfide bonds. In order to investigate the biological role of this Cys37 in VPAC1, the wild-type VPAC1 and Cys37/Ala mutant (VPAC1-C37/A) were expressed stably as fusion proteins with enhanced yellow fluorescent protein (EYFP) respectively in Chinese hamster ovary (CHO) cells. Both VPAC1-EYFP and VPAC1-C37/A-EYFP trafficked to the plasma membrane normally, and CHO cells expressing VPAC1-EYFP displayed higher anti-apoptotic activity against camptothecin (CPT) induced apoptosis than the cells expressing VPAC1-C37/A-EYFP, while VPAC1-C37/A-CHO cells showed higher proliferative activity than VPAC1-CHO cells. Confocal microscopic analysis, western blotting and fluorescence quantification assay showed VPAC1-EYFP displayed significant nuclear translocation while VPAC1-C37/A-EYFP did not transfer into nucleus under the stimulation of VIP (0.1 nM). Acyl-biotin exchange assay and click chemistry-based palmitoylation assay confirmed for the first time the palmitoylation of Cys37, which has been predicted by bioinformatics analysis. And the palmitoylation inhibitor 2-bromopalmitate significantly inhibited the nuclear translocation of VPAC1-EYFP and its anti-apoptotic activity synchronously. These results indicated the palmitoylation of the Cys37 in the N-terminal extracellular domain of VPAC1 mediates the nuclear translocation of VPAC1 contributing to its anti-apoptotic activity. These findings reveal for the first time the lipidation-mediating nuclear translocation of VPAC1 produces a novel anti-apoptotic signal pathway, which may help to promote new drug development strategy targeting VPAC1.

## INTRODUCTION

The receptor VPAC1 belonging to class B G-protein coupled receptors (GPCR) family is shared by pituitary adenylate cyclase activating polypeptide (PACAP) and vasoactive intestinal polypeptide (VIP) with identical binding affinity [1]. VPAC1 not only localizes in central nervous system such as forebrain, thalamus, cerebral cortex and hippocampus, but also spreads in numerous peripheral organs, including liver, kidney, prostate, breast, spleen, lung, gastrointestinal tract, and in almost all epithelial tissues [2]. Moreover, the expression of VPAC1 is also reported associated with some special physiological and pathological processes [3]. For example, high expression level of VPAC1 has been found in various malignant epithelia-derived tumors and their metastases, such as colorectal carcinoma [4], breast carcinoma [5], prostate carcinoma [6] and lung carcinoma. On the other hand, the lower expression level of VPAC1 is corresponding to the more severe inflammation and the higher disease activity in rheumatoid arthritis [7, 8]. Since the variation of VPAC1 expression is always correlated with the occurrence and development of diseases such as tumor and immune disorders, the structure-activity-relation study of VPAC1 not only help to explain the physiological and pathological functions of VPAC1, but also provide the research basis for the drug screening and development targeting VPAC1 against tumors and autoimmune disorders.

**Figure 1:**
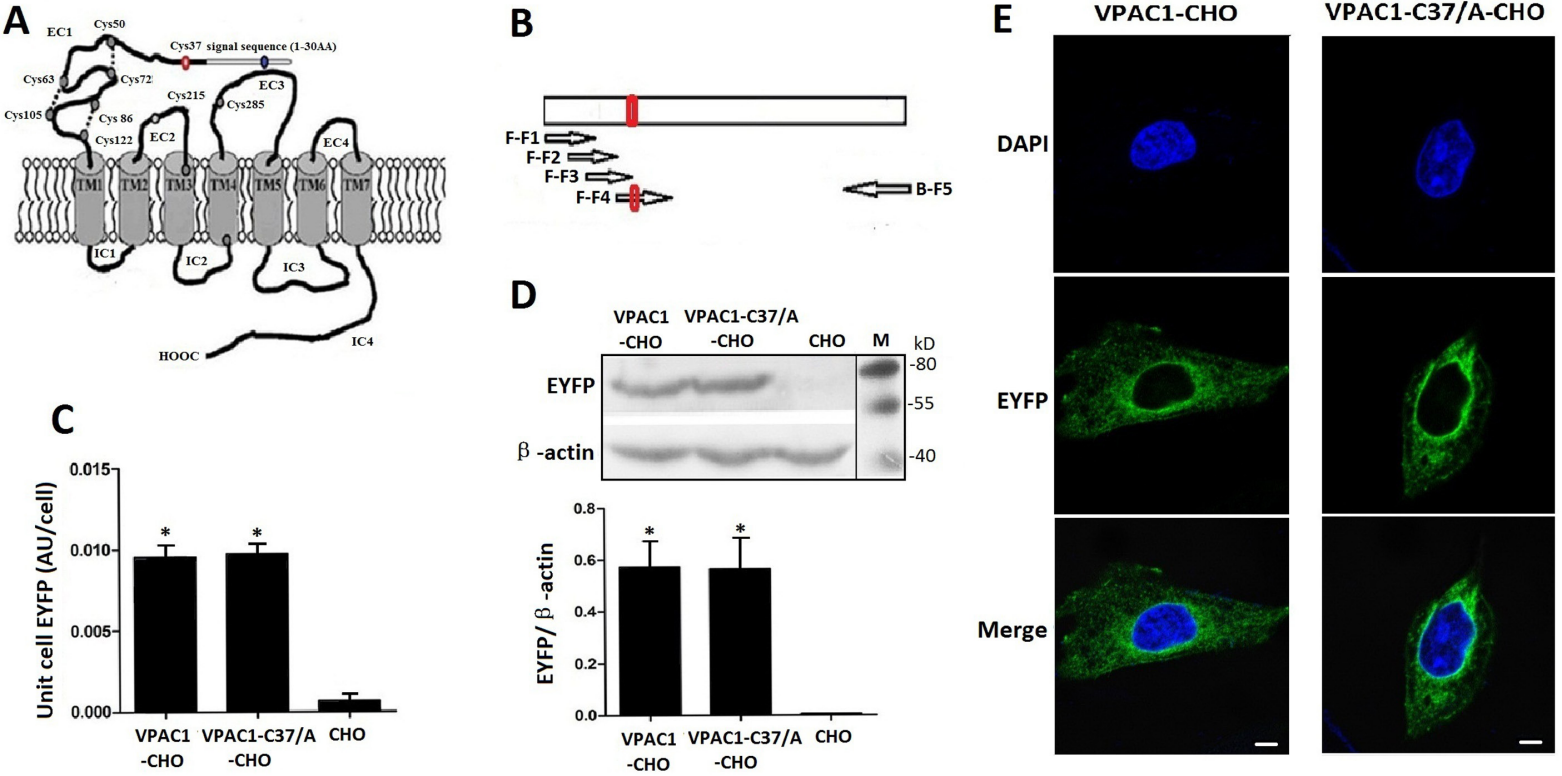
The structure draft of VPAC1 (**A**), the construction of the gene encoding VPAC1-C37/A (**B**) and the expression (**C**, **D**) and the plasma membrane trafficking (**E**) of VPAC1-EYFP and VPAC1-C37/A-EYFP in CHO cells. A The structure draft of VPAC1. The red circle indicates the Cys37. B The construction of the gene encoding VPAC1-C37/A amplified using overlapping PCR (The arrows represented the primers). The red circle indicates the mutation coding Cys37/A. C Fluorescence quantification assay using the multi-label counter with excitation (460 ± 30 nm) and emission (535 ± 30 nm) showed that VPAC1-CHO had equal receptor EYFP fluorescence densities with VPAC1-C37/A-CHO, which indicated that the expression levels of VPAC1-EYFP in CHO cells were equal to those of VPAC1-C37/A-EYFP (*P < 0.01, vs. CHO). The data were means ± SEM of four experiments. D Western blotting results showed that VPAC1-EYFP and VPAC1-C37/A-EYFP had equal expression levels in CHO cells. The data were means ± SEM of four experiments. E Confocal fluorescence imaging showed that both VPAC1-EYFP and VPAC1-C37/A-EYFP transported to the cell surface normally. Bar. 5 μm.

The class B GPCRs as receptors of peptide hormones have conserved large N-terminal extracellular domain with relatively complex structure characterized by three conserved intramolecular disulfide bonds, which is significantly different from other GPCR classes [9]. Except the signal peptide, the N-terminal extracellular domain of mature VPAC1 has 7 cysteines (Cys), of which, three pairs of intramolecular disulfide bonds conserved in class B GPCR are formed by Cys50-Cys72, Cys63-Cys105 and Cys86-Cys122 respectively [10]. Cys37 in mature VPAC1 is a free cysteine residue not involved in the formation of any disulfide bonds reported (as show in Figure 1A). Our previous report has shown the mutation of the similar first free Cys in N-terminal extracellular domain of PAC1, which is PACAP preferring receptor with high homology with VPAC1 also belonging to class B GPCRs, significantly inhibited the anti-apoptotic activity mediated by PAC1 [11]. So it attracted our interest to figure out the role of the first free Cys37 in the N-terminal extracellular domain of VPAC1 played in the profiles of VPAC1.

In this research, the first free Cys37 was mutated to Ala to construct the mutant VPAC1-Cys37/Ala (VPAC1-C37/A). We constructed the Chinese hamster ovary (CHO) cells with high expression of wild type VPAC1 and mutant VPAC1-C37/A fused with enhanced yellow fluorescent protein (EYFP) respectively and detected the role of Cys37 in the anti-apoptotic activity mediated by VPAC1 using camptothecin (CPT) induced apoptosis. CPT is accepted as a plant anticancer drug targeting topoisomerase I, which has been confirmed to induce apoptosis of various tumor cells including breast cancer, leukemia, lung cancer, liver cancer, stomach cancer, etc [12]. It was found for the first time in this research that the palmitoylation of Cys37 mediates the nuclear translocation of VPAC1 contributing to the VPAC1-mediating anti-apoptotic activity.

## RESULTS

### High expression of VPAC1-EYFP and VPAC1-C37/A-EYFP in CHO cells

Fluorescence quantification of the whole cellular protein (Figure 1C) and western blotting of the whole cellular protein (Figure 1D) were performed to determine the stable expressions and equal levels to each other of VPAC1-EYFP and VPAC1-C37/A-EYFP in CHO cells. Fluorescence confocal microscope images showed that both VPAC1-EYFP and VPAC1-C37/A-EYFP trafficked to the plasma membrane (Figure 1E). Furthermore, after the data from [^125^I]-VIP competition binding assay using the cell membrane fraction was calibrated by the EYFP fluorescence density of the cell membrane fraction representing the EYFP-tagged receptor density in the cell membrane fraction, it was shown that VPAC1-C37/A-EYFP had dissociation constant (Kd) of 0.42 ± 0.07 nM and binding capacity (Bmax) of 0.49 ± 0.07 (pmol/mg fluorescent protein) with VIP, which was almost equal to VPAC1-EYFP with Kd of 0.39 ± 0.08 nM and Bmax of 0.52 ± 0.09 (pmol/mg fluorescent protein). So we considered that the mutation of Cys37/Ala in the N-terminal extracellular domain did not interference the binding of the receptor with its ligand VIP. The construction of VPAC1-CHO cell line and VPAC1-C37/A-CHO cell line laid the foundation for the subsequent research.

### Proliferative activities of VPAC1-CHO cells and VPAC1-C37/A-CHO cells

**Figure 2:**
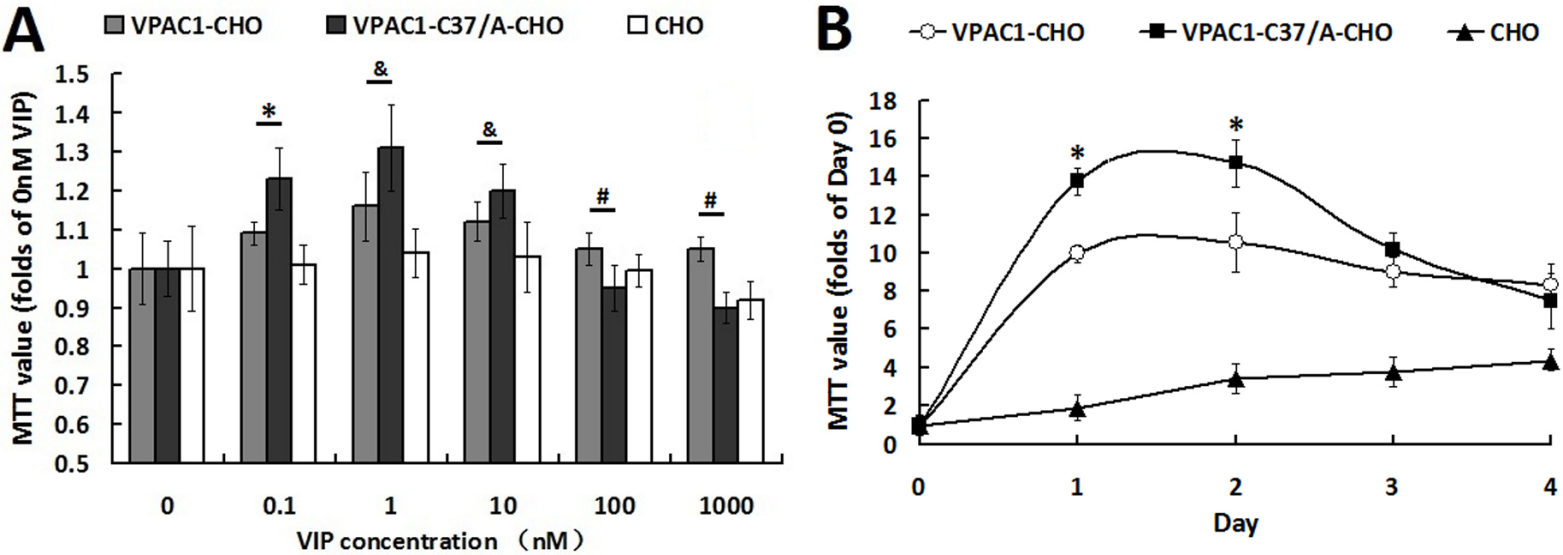
VPAC1-CHO cells had lower proliferative activity than VPAC1-C37/A-CHO cells (**A**) The cell viabilities assayed by MTT methods of VPAC1-CHO, VPAC1-C37/A-CHO and CHO cells incubated with VIP (0.1 nM–1000 nM). VPAC1-C37/A-CHO cells proliferated more rapidly than VPAC1-CHO cells when incubated with low concentration of VIP (0.1 nM–10 nM) (*p < 0.01, VPAC1-C37/A-CHO vs. VPAC1-CHO; ^&^*p* < 0.05, VPAC1-C37/A-CHO vs. VPAC1-CHO), but VPAC1-CHO cells remained higher viability than VPAC1-C37/A-CHO cells when incubated with high concentration of VIP (100 nM–1000 nM) (^#^*p* < 0.01, VPAC1-CHO vs. VPAC1-C37/A-CHO). The data were means ± SEM of six experiments. (**B**) The growth curve of VPAC1-CHO, VPAC1-C37/A-CHO and CHO cells with 0.1 nM VIP for 4 days. VPAC1-C37/A-CHO cells proliferated more rapidly than VPAC1-CHO cells before the logarithmic phase (*p < 0.01, VPAC1-C37/A-CHO vs. VPAC1-CHO), but faded more rapidly than VPAC1-CHO cells after the logarithmic phase. The data were means ± SEM of six experiments.

The assay on the proliferative activities induced by VIP (Figure 2A) showed that VPAC1-C37/A-CHO cells proliferated significantly more rapidly than VPAC1-CHO cells when incubated with low concentration rage of (0.1 nM–10 nM) VIP. As a peptide hormone, VIP always displays the hormesis effect, which may be the reason why VIP in much high concentration range shows negative scathing effect on the cells viability. VPAC1-CHO cells remained higher viability than VPAC1-C37/A-CHO cells under higher concentrations of VIP (100 nM–1000 nM) indicating that VPAC1-CHO cells had higher anti-apoptotic activity than VPAC1-CHO cells.

And when the cells were incubated with 0.1 nM VIP for 4 days, it was shown by the growth curves (Figure 2B) that VPAC1-C37/A-CHO proliferated significantly more rapidly than VPAC1-CHO cells in the logarithmic phase. But behind the logarithmic phase, the viability of VPAC1-C37/A-CHO cells declined more rapidly than that of VPAC1-CHO cells. These results indicated that VPAC1-C37/A-EYFP mediated more proliferative viability but less anti-apoptotic activity than VPAC1-EYFP.

### Anti-apoptotic activity mediated by VPAC1-EYFP and VPAC1-C37/A-EYFP against CPT

**Figure 3:**
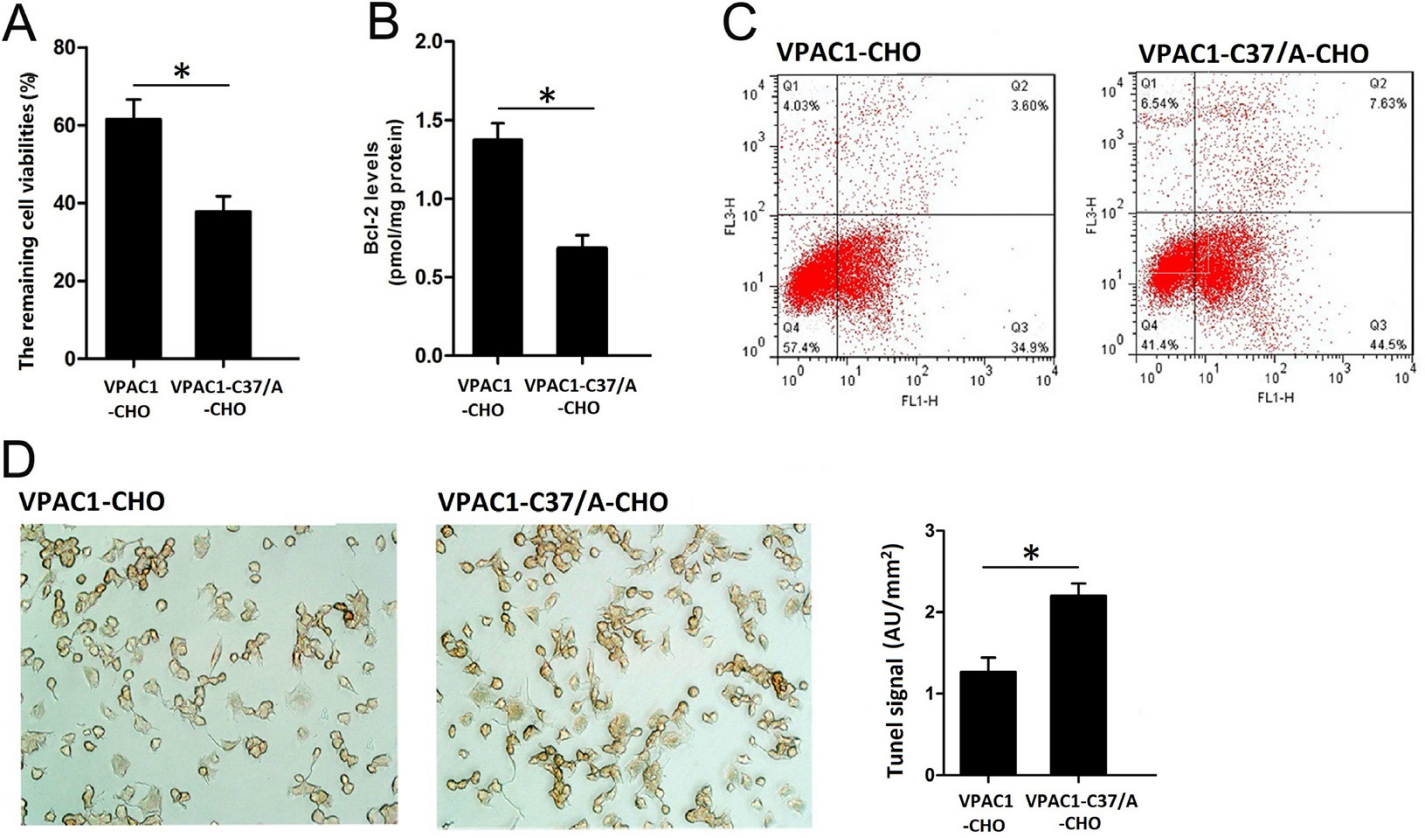
VPAC1-CHO cells had higher anti-apoptotic activity than VPAC1-C37/A-CHO cells against CPT induced apoptosis (**A**) The remaining cells viabilities after VIP incubation and CPT induced apoptosis by MTT assay showed that the viabilities remained of the VPAC1-CHO cells was significantly higher than that of VPAC1-C37/A-CHO cells (*P < 0.01, VPAC1-CHO vs. VPAC1-C37/A-CHO). The data were means ± SEM of six experiments. (**B**) The plasma anti-apoptotic protein Bcl-2 levels after VIP incubation and CPT induced apoptosis showed that VPAC1-CHO cells had significant higher Bcl-2 levels than VPAC1-C37/A-CHO cells (*P < 0.01, VPAC1-CHO vs. VPAC1-C37/A-CHO). The data were means ± SEM of six experiments. (**C**) The anti-apoptotic viabilities in VPAC1-CHO and VPAC1-C37/A-CHO by flow cytometry assay in CPT induced apoptosis showed that the apoptotic percentage of VPAC1-CHO (3.6% in Q3 quadrant) is significantly lower than that of VPAC1-C37/A-CHO (7.63% in Q3 quadrant). (**D**) Microscope imaging of VPAC1-CHO and VPAC1-C37/A-CHO cells treated with both VIP (0.1 nM) and CPT (50 uM) and stained by TUNEL assay. It was shown that brown staining in VPAC1-CHO cells was significantly less than VPAC1-C37/A-CHO cells. The statistic analysis on the TUNEL signals showed that VPAC1-CHO cells had significant lower TUNEL signal than VPAC1-C37/A-CHO cells (*P < 0.01, VPAC1-CHO vs. VPAC1-C37/A-CHO.) The data were means ± SEM of four experiments.

The MTT assay results shown in Figure 3A indicated that VPAC1-CHO cells pre-incubated with 0.1 nM VIP for 0.5–1 h had the remaining cell viability of 62.3 ± 6.2% after the treatment with CPT for 12 h, which was about 1.7 fold higher than VPAC1-C37/A-CHO cells with the remaining cell viability of 38.2 ± 4.2%. And the level of anti-apoptosis protein Bcl-2 in VPAC1-CHO cells was significantly higher than that in VPAC1-C37/A-CHO cells (Figure 3B). The flow cytometry results also showed that after the pre-treatment with 0.1nM VIP, the apoptotic percentage of VPAC1-CHO cells was significantly lower than that of VPAC1-C37/A-CHO cells (Figure 3D). The results of Tunel assay showed that the brown stained cells of VPAC1-CHO were significantly less than that of VPAC1-C37/A-CHO (Figure 3E). After the Tunel signals were quantified, it was shown that the Tunel signal of VPAC1-CHO cells was significantly weaker than that of VPAC1-C37/A-CHO cells (Figure 3E).

All above results indicated that activation of VPAC1 produced stronger anti-apoptotic activity against CPT-induced apoptosis than VPAC1-C37/A.

### Translocation of VPAC1-EYFP and VPAC1-C37/A-EYFP in CHO cells induced by VIP

**Figure 4:**
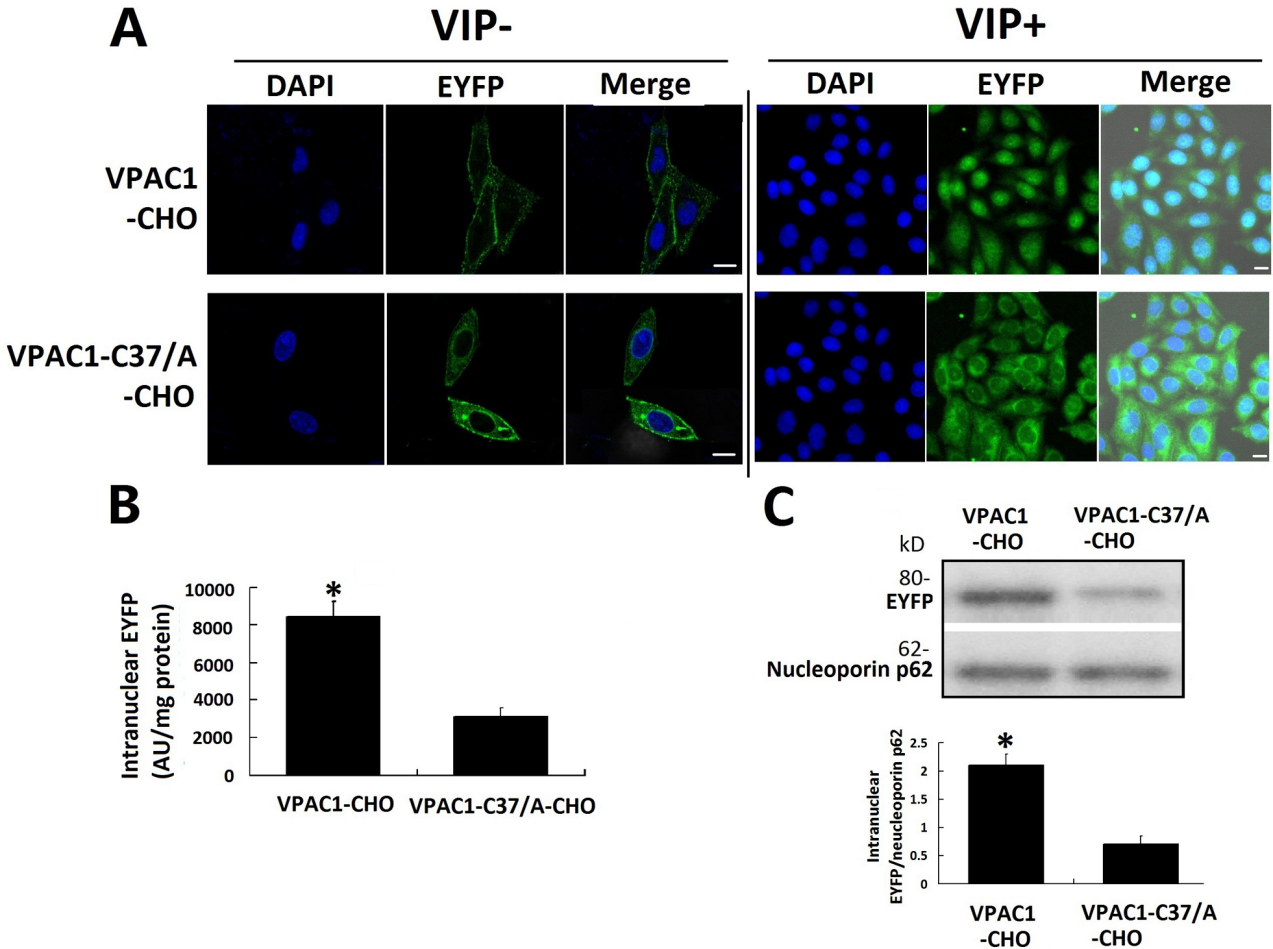
VPAC1-EYFP trans-localized into the nucleus induced by VIP (**A**) Confocal fluorescence micrographs showed that VPAC1-EYFP and VPAC1-C37/A-EYFP transported normally onto plasma membrane. After the treatment with VIP, most of fluorescence signal representing VPAC1-EYFP trans-localized from cell surface into nucleus, while most VPAC1-C37/A-EYFP internalized but outside the nucleus and did not trans-localized into nucleus. Bar, 5 μm. (**B**) Intranuclear fluorescence quantification showed the intranuclear fluorescence signal in VPAC1-CHO cells was significantly stronger than that in VPAC1-C37/A-EYFP (*P < 0.01, VPAC1-CHO vs. VPAC1-C37/A-CHO). The data were means±SEM of six experiments. (**C**) Western blotting results of nucleoprotein showed that the amount of VPAC1-EYFP localized in the nucleus was much larger than the intranuclear amount of VPAC1-C37/A-EYFP (*P < 0.01, VPAC1-CHO vs. VPAC1-C37/A-CHO). The data were means ± SEM of four experiments.

The fluorescence confocal microscope images showed that after incubating the cells with 0.1 nM VIP for 1 h, the intracellular translocations of VPAC1-EYFP and VPAC1-C37/A-EYFP i were significantly different from each other. Most of the EYFP signal in VPAC1-CHO cells was shown to locate inside the nucleus, while the green EYFP signal in the VPAC1-C37/A-CHO cells mainly distributed outside the nucleus in the plasma (Figure 4A). The fluorescence quantification (Figure 4B) showed that the intranuclear EYFP signal intensity inVPAC1-CHO cells was s about 3 folds of that in VPAC1-C37/A-CHO cells, which was consistent with the result of western blotting of intranuclear EYFP (Figure 4C). All these assays confirmed the nuclear translocation of VPAC1-EYFP induced by VIP and the mutation of C37/A significantly interfered the receptor's nuclear translocation after its activation. It was estimated that the nuclear translocation of VPAC1 may be associated with the anti-apoptotic activity mediated by VPAC1.

### Bio-information prediction of modification on Cys37 of VPAC1

**Figure 5:**
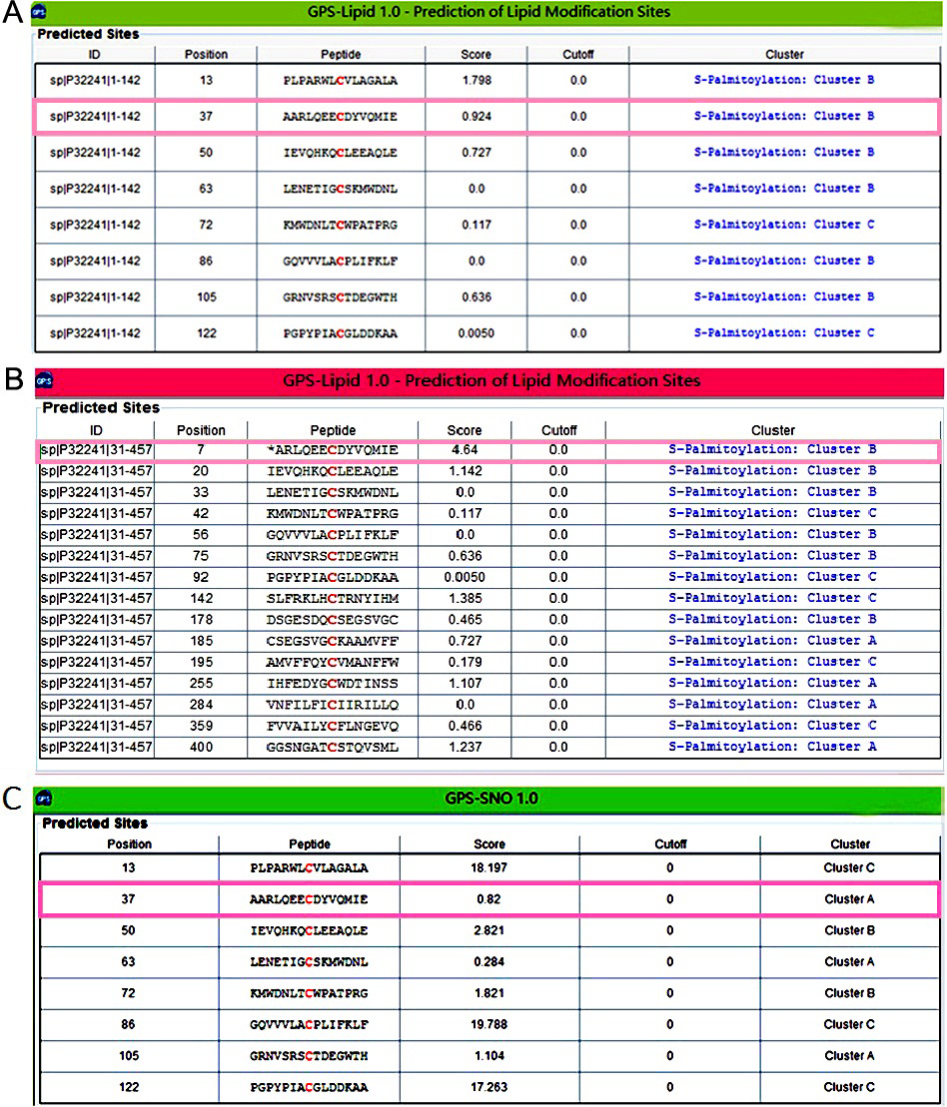
The bio-information analysis indicated the most palmitoylation possibility on Cys37 of VPAC1 (**A**) Shown was the prediction result of lipid modification on the cysteines in the 1-142 aa (with signal peptide) N-terminal extracellular domain of VPAC1. The score of Cys37 was indicated in pink frame. (**B**) Shown was the prediction result of lipid modification on the cysteines in the 31-457 aa (without signal peptide) mature VPAC1. The score of Cys37 was indicated in pink frame. (**C**) Shown was the prediction result of nitrosylation on the cysteines in the 1-142 aa (with signal peptide) *N*-terminal extracellular domain of VPAC1. The score of Cys37 was indicated in pink frame.

After the 1-142 aa extracellular domain (with signal peptide) and the 31-457 aa mature VPAC1 (without signal peptide) were submitted to the prediction of modification on the cysteines. Since the higher score value indicates the more modification potential, in the 1-142 aa extracellular domain, the score of Cys37 for palmitoylation is 0.924 just behind Cys13 in the signal peptide (Figure 5A), while the score for palmitoylation of Cys37 in 31-457 aa mature VPAC1 is the highest (Figure 5B). As for nitrosylation, the score of Cys37 is 0.82 significant lower than the other 7 cysteines except Cys63 in the 1-142 aa extracellular domain (Figure 5C). The bio-information analysis results indicated that Cys37 has high possibility to be subjected to S-palmitoylation but not to nitrosylation.

### Confirmation of S-palmitoylation of Cys37 of VPAC1 by acyl-biotin exchange assay and click chemistry palmitoylation assay

**Figure 6:**
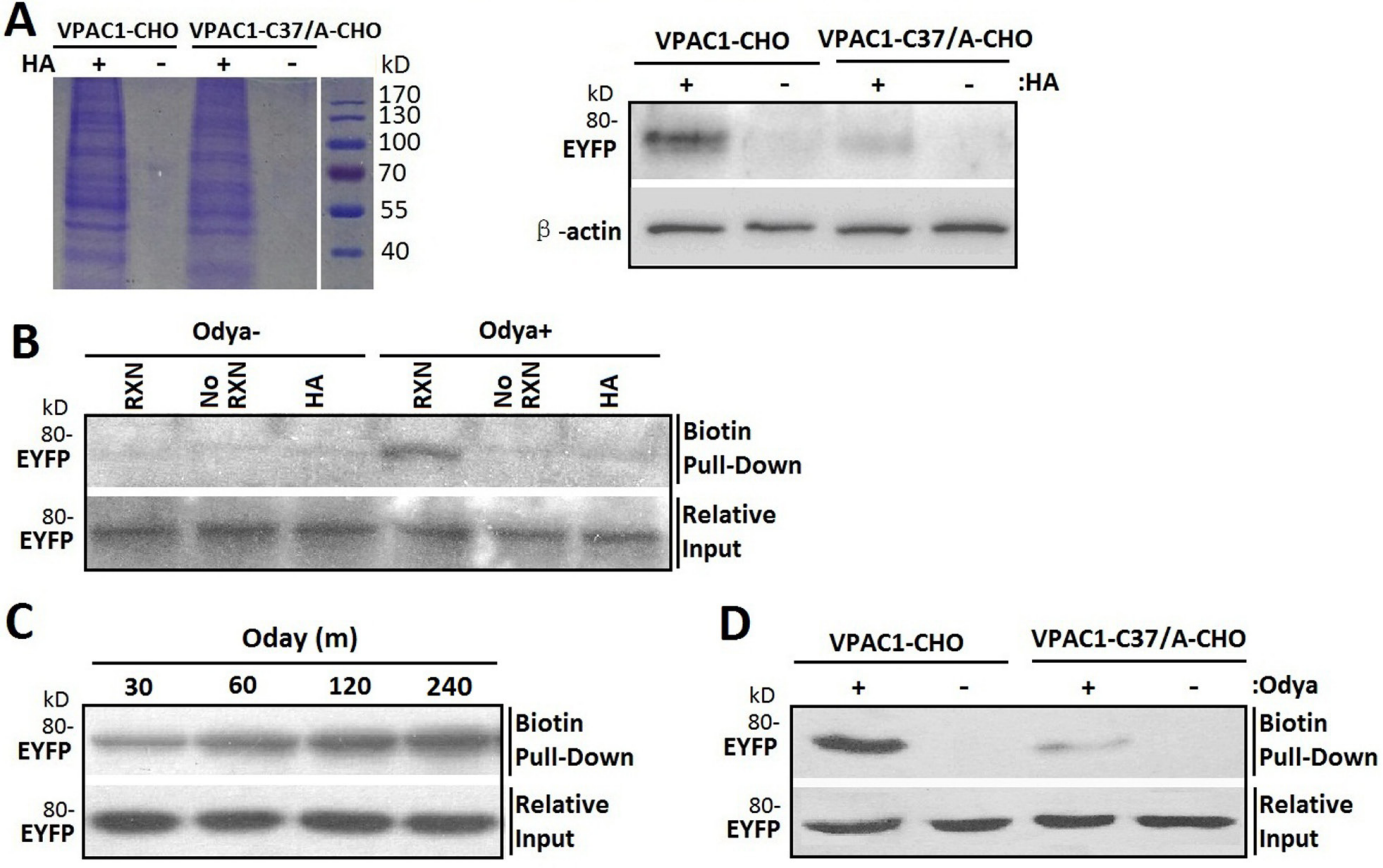
Cys37 in VPAC1 is S-palmitoylated determined by acyl-biotin exchange assay and click chemistry palmitolaytion assay (**A**) After the biotinylated proteins from VPAC1-CHO and VPAC1-C37/A-CHO subjected to the acyl-biotin exchange assay with (HA+) or without (HA-) hydroxylamine treatment were pulled down by streptavidin-agarose and electrophoresed by SDS-PAGE (left) and then detected using anti-EYFP antibody (right), VPAC1-EYFP was significantly detectable, while VPAC1-C37/A-EYFP is almost undetectable, indicating that Cys37 was palmitoylated. And the negative interference of HA showed that the acyl-biotin exchange assay was HA depended and the palmitoylation of Cys37 is via hydroxylamine-sensitive thioester bond. (**B**) VPAC1-CHO cells were incubated for 12 hours with (Odya+) or without (Odya-) palmitate ortholog. Samples were divided and either not biotinylated through the click reaction (No RXN), reduced prior to the reaction with HA, or biotinylated and not reduced (RXN). Only samples treated with RXN without HA showed significant signal, supporting the specificity of the click chemistry palmitolaytion assay. Relative equal inputs without click chemistry palmitolaytion assay were detected using anti-EYFP antibody. (**C**) After VPAC1-CHO cells were incubated with Odya for 30, 60, 120 and 240 min hours, click reaction was performed followed by biotin pull-down and western blot analysis for EYFP. The time-dependent signals confirmed the technique's validity. Relative equal inputs without click chemistry palmitolaytion assay were detected using anti-EYFP antibody. (**D**) After VPAC1-CHO cells and VPAC1-C37/A-CHO cells were incubated with (Odya+) or without (Odya-) palmitate ortholog, click reaction was performed followed by biotin pull-down and western blot analysis for EYFP. VPAC1-EYFP was significant detectable, while VPAC1-C37/A-EYFP was almost undetectable, indicating the palmitoylation of Cys37 was determined by the click chemistry palmitolaytion assay. Relative equal inputs without click chemistry palmitolaytion assay were detected using anti-EYFP antibody. Representative blots from at least three independent experiments are shown.

To test whether Cys37 is palmitoylated, we utilized acyl-biotin exchange technique for labeling cysteine residues modified via a thioester bond like S-palmitoylation with biotin depending on hydroxylamine (HA) cleavage. In brief, after blocking free sulfhydryls with N-ethylmaleimide (NEM), treatment with HA cleaved the thioester bond between palmitate and cysteine residue, exposing the bound thiol, which is then covalently linked to biotin-HPDP. The biotinylated proteins were purified by streptavidin-agarose. The SDS-PAGE of the elution from streptavidin-agarose column showed that the biotinylation is dependent on the cleavage of HA of the thioester bonds of S-palmitoylation (Figure 6A left). And the subsequent western blotting using monoclonal antibody recognizing EYFP showed that VPAC1-CHO had stronger posotive signal than VPAC1-C37/A-CHO (Figure 6A right) indicating that the Cys37 was subjected to S-palmitoylation.

Next we used click chemistry palmitoylation assay to confirm the palmitloylation of Cys37. In brief, the palmitoylated cystine was metabolic labeled with palmitate ortholog alkyne-linked 17-Octadecynoic acid (Odya), which was then covalently cross-linked with biotin by the click chemical reaction between alkyne-linked 17-Odya and biotin-azide. The biotinylated proteins were pulled-down using streptavidin-agarose, and further submitted to immunoblotting for EYFP. To test the reaction specificity, the cell lysates of VPAC1-CHO metabolic labeled with Odya were divided into those processed through the click-reaction (RXN) versus those not (No RXN) and samples processed with HA to remove any cysteine-linked Odya. The subsequent western blotting revealed VPAC1-EYFP pull-down was dependent on the click-reaction and was prevented by reducing thioester bonds agent HA, again supporting the S-palmitoylation of VPAC1-EYFP (Figure 6B). Moreover the biotin-labeling of VPAC1 exhibited time dependence (Figure 6C), supporting the technique's validity.

Compared to VPAC1-CHO, VPAC1-C37/A-CHO showed significant weaker biotin-labeling signal (Figure 6D), indicating that the Cys37 offers an effective palmitoylation site. The remaining signals of VPAC1-C37/A-CHO in both acyl-biotin exchange assay and click chemistry palmitoylation assay indicated that there are other palmitoylation sites except Cys37 in VPAC1, such as Cys13 in the signal peptide of VPAC1 estimated by bio-information analysis.

### The synchronous inhibitory effects of 2-BP showed the nuclear translocation of VPAC1 contributed to the anti-apoptotic activity of VPAC1

**Figure 7:**
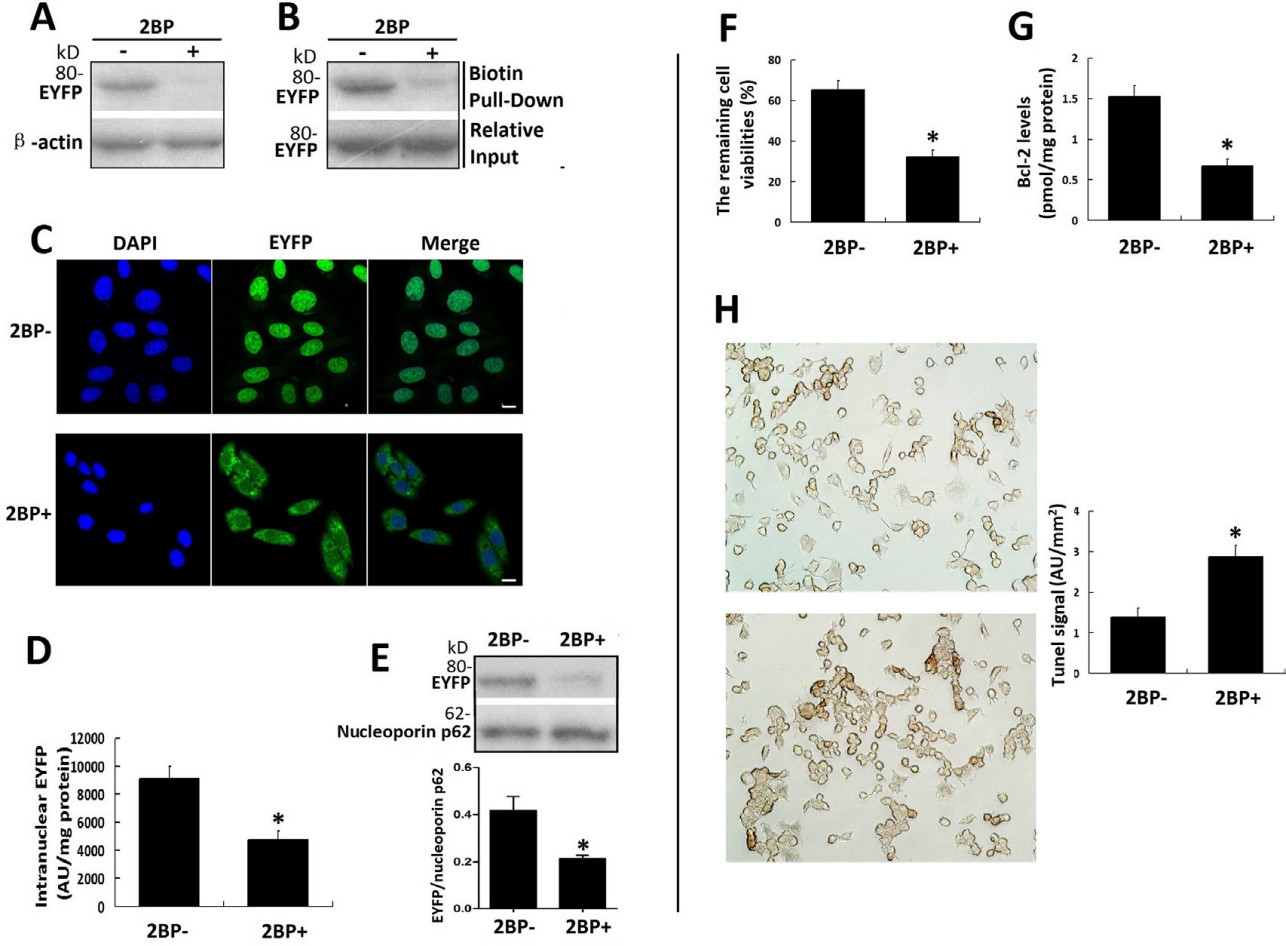
Palmitoylation inhibitor 2-BP inhibited the nuclear translocation of VPAC1-EYFP and the anti-apoptotic activity of VPAC1-CHO cells synchronously (**A**) The acyl-biotin exchange assay was used to confirm the inhibitory effects of 2-BP on the palmitoylation of VPAC1-EYFP. (**B**) The click chemistry palmitolaytion assay was used to confirm the inhibitory effects of 2-BP on the palmitoylation of VPAC1-EYFP. (**C**) Confocal fluorescence micrographs showed the fluorescence signal representing VPAC-EYFP internalized but did not further transfer into nucleus while incubated with 2-BP, indicating that 2-BP inhibited the nucleus translocation of VPAC1-EYFP induced by VIP. Bar, 5 μm. (**D**) Intranuclear fluorescence quantification showed the intranuclear fluorescence signal of VPAC1-EYFP in treatment with 2-BP (2BP+) was significantly lower that that in treatment with without 2-BP (2BP-) (*P < 0.01, 2BP+ vs. 2BP-). The data were means ± SEM of six experiments. (**E**) Western blotting of nucleoprotein showed that the amount of the intranuclear VPAC1-EYFP was significantly decreased by treatment with 2-BP (*P < 0.01, 2BP+ vs. 2BP-). The data were means ± SEM of six experiments. (**F**) The treatment with 2-BP decreased the percentage of the cell remaining viability of VPAC1-CHO cells (*P < 0.01, 2BP+ vs. 2BP-). The data were means ± SEM of six experiments. (**G**) The treatment with 2-BP decreased the intracellular anti-apoptotic protein Bcl-2 level of VPAC1-CHO cells. (*P < 0.01, 2BP+ vs. 2BP-). The data were means ± SEM of six experiments. (**H**) Microscope imaging of VPAC1-CHO cells treated with 2-BP (2BP+) or without 2-BP (2BP-) were dyed by TUNEL assay. It was shown that brown staining in VPAC1-CHO cells treated with 2-BP was significantly more than that treated without 2-BP, which was confirmed by the statistic analysis on the TUNEL signals (*P < 0.01, 2BP+ vs. 2BP-). The data were means ± SEM of six experiments.

The palmitate analog 2-bromopalmitate (2-BP) is most commonly used as palmitoylation inhibitor in cells. After the inhibitory effect of 2-BP on the palmitoylation of VPAC1-EYFP was confirmed by acyl-biotin exchange assay and click chemistry palmitoylation assay (Figure 7A), fluorescence confocal microscope imaging (Figure 7B), intranuclear fluorescence quantification (Figure 7C) and western blotting of intranuclear VPAC1-EYFP (Figure 7D) showed that the treatment with 2-BP inhibited the nuclear translocation of VPAC1-EYFP induced by VIP significantly. Meanwhile the anti-apoptotic activity mediated by VPAC1-EYFP was also significantly inhibited synchronously by 2-BP, as shown by the decreased remaining cell viability (Figure 7E), the lowered intracellular Bcl-2 level (Figure 7F) and increased apoptotic signal in flow cytometry (Figure 7G).

These results indicated that the palmitoylation of Cys37 mediates the the nuclear translocation after its activation, which contributes to the anti-apoptotic activity mediated by VPAC1.

## DISCUSSION

In this research, we firstly confirmed that the mutation of Cys37/A dose not influence the receptor's cell membrane trafficking and its binding with VIP, which is consistent with the previous report by Gaudin et al. [16]. The constructions of VPAC1-CHO cell line and VPAC1-C37/A-CHO cell line with similar receptors expression levels lay the foundation for the subsequent research on the role of Cys37 in the profiles of VPAC1.

It was found for the first time in this research that CHO cells with high expression of native VPAC1-EYFP displayed higher anti-apoptotic activity than cells with high expression of mutant VPAC1-C37/A-EYFP under the activation of VIP (0.1nM), while VPAC1-C37/A-CHO cells proliferated more rapidly than VPAC1-CHO cells in normal growth condition. It was also observed that the nuclear translocation of VPAC1-EYFP was significantly much more dominant than the nuclear translocation of VPAC1-C37/A-EYFP after the receptors’ activation by VIP (0.1nM), indicating that Cys37 mediates the nuclear translocation of VPAC1-EYFP. After the bio-information analysis showed the high possibility of the palmitoylation of Cys37, the acyl-biotin exchange assay and click chemistry-based palmitoylation assay were both confirmed the palmitoylation of Cys37. And the palmitoylation inhibitor 2-BP not only inhibited the nuclear translocation of VPAC1-EYFP, but also inhibited its anti-apoptotic activity synchronously. All these findings suggested for the first time that palmitoylation of Cys37 mediates the nuclear translocation of VPAC1 after its activation, which contributes to the anti-apoptotic activity mediated by VPAC1.

It was found for the first time in this research by fluorescence confocal micrographs, fluorescence quantification and western blotting of intranuclear EYFP-tagged receptors that VPAC1 translocates to the nucleus after its activation by VIP. The atypical compartmentalization of GPCRs at the cell nucleus has been reported to play different functional roles such as in gene expression from GPCRs the on plasma membrane [17]. And there have been several reports about the nucleus location of VPAC1 in tumor cells. Cases in point, the analysis of nuclear staining revealed with up to 50% of human glioblastoma cells displayed strong VPAC1 nuclear staining, whereas the nuclear VPAC2 staining remained marginal; and the significant increase of VPAC1 staining is positively associated with the glioma grade [18]. Moreover, the marked nuclear localization of VPAC1 is also found in human colonic adenocarcinoma cell line [19], estrogen-dependent (T47D) and independent (MDA-MB-468) human breast cancer cell lines [20]. As for our study, the CHO cells with high expression of VPAC1 displayed effective anti-apoptotic activity against CPT induced apoptosis associated the nucleus translocation of VPAC1 after its activation by VIP, indicating the nuclear translocation of VPAC1 plays an functional role in the formation of the anti-apoptotic activity of VPAC1. Similar phenomena have been reported, such as sphingosine-1-phosphate receptor subtype 1 (S1P(1)), a GPCR, which is also submitted to ligand-induced nuclear translocation. And the nuclear S1P(1) is able to regulate the transcription of Cyr61 and CTGF, two growth factors functionally important in the regulation of vasculature [21]. Moreover the nuclear localized GPCRs have been indicated to modulate nuclear ionic homeostasis and function. And these nuclear membrane GPCRs exert functions independent on the plasma membrane GPCRs to contribute to protein synthesis and undergo changes in pathological conditions [22]. So the nuclear translocation of VPAC1 induced by VIP, to our opinion, is involved in the formation of the anti-apoptotic activity of VPAC1 maybe by contributing to the significant up-regulation of intracellular Bcl-2. The functional role of the intra-nuclear VPAC1 and its role in the anti-apoptotic activity do need further more detailed research.

Furthermore, we considered the nuclear translocation of VPAC1 after its activation makes VPAC1 have slower turnover efficiency than VPAC1-C37/A after its internalization and makes VPAC1 not re-localize to the plasma membrane as efficiently and rapidly as VPAC1-C37/A. So the higher turnover efficiency and more rapid re-localization of VPAC1-C37/A to the plasma membrane without nuclear translocation make the VPAC1-C37/A-CHO cells more sensitive to VIP and proliferate more rapidly than VPAC1-CHO in normal growth condition. But without the nuclear translocation, which produces the anti-apoptotic signal pathways, VPAC1-C37/A-CHO cells faded more rapidly than VPAC1-CHO after rapid growth phase, and had lower viability under VIP in high concentration range (100 nM–1000 nM). The negative effects of VIP in high concentration range have been reported, which to our opinion belong to the hormesis effects mediated by the peptide hormones.

In addition, we consider the palmitoylation of the first free Cys37 of VPAC1, which was firstly estimated by bio-information analysis and further confirmed by experiments for the first time in our study played a key role in profiles of VPAC1. As we known, the palmitoylation aid GPCRs’ localization in some specific lipid raft, that has been considered to contribute to the oligomerization of GPCRs [23], lead to the interaction of GPCRs with other membrane proteins, then influence the signal transduction after the activation of GPCRs and affect the GPCRs’ fates following activation including proteolysis or recycle or translocation [24]. A group of reports have indicated the palmitoylation help the nuclear localization of some protein. Cases in point, G protein coupled receptor kinase 6A displays nuclear translocation depending on palmitoylation [25]; palmitoylation of cytoskeleton-associated protein-4 regulates its nuclear translocation and DNA binding [26]; and palmitoylation regulates plasma membrane and nuclear shuttling of R7BP (a novel membrane anchor for the RGS7 family) [27]. So we have reason to consider it is the palmitoylation of Cys37 that mediated the nuclear translocation of VPAC1 after its activation.

**Figure 8:**
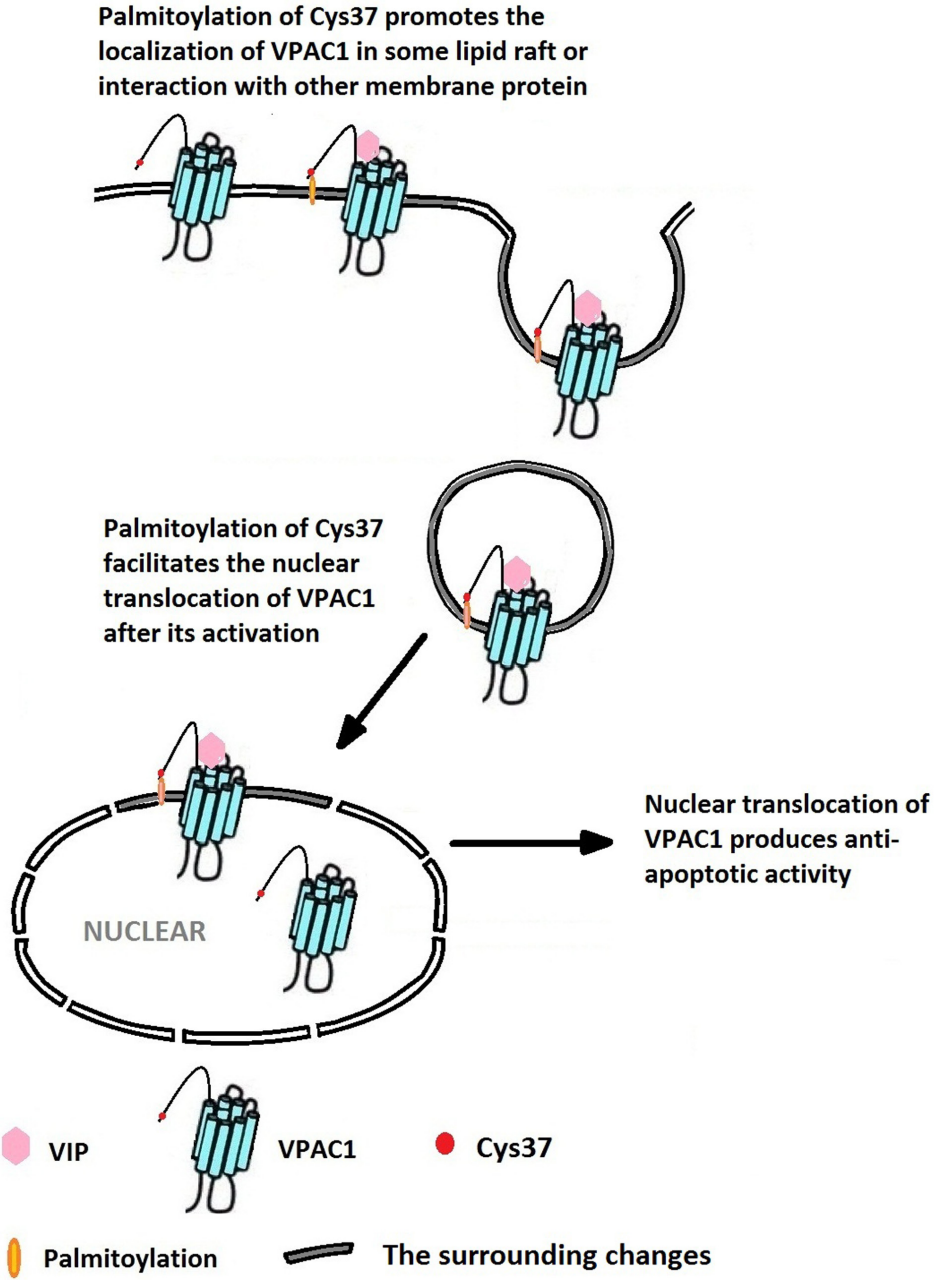
The palmitoylation of Cys37 plays a key role in the nuclear translocation of VPAC1 which is involved in the formation of the anti-apoptotic activity mediated by VPAC1 after its activation by VIP

To our opinion, the palmitoylation of the first Cys37 in the N-terminal extracellular domain of VPAC1 promotes the localization of VPAC1 in some kinds of lipid raft, or induces the interaction or the oligomerization of VPAC1 with some membrane proteins, and finally contributes to the nuclear translocation of VPAC1. In a conclusion, as shown in Figure 8, the lipidation of the extracellular Cys37 mediates the nuclear translocation of VPAC1 after its activation, which contributes to a novel anti-apoptotic signal pathway. These findings not only help us to understand the physiological and pathological functions of VPAC1 more deeply, but also promote the drug development targeting GPCR VPAC1. Cases in point, the interference of the lipidation or the nuclear translocation of VPAC1 may help to cure the tumor with high expression of VPAC1. On the contrary, the protective functions mediated by VPAC1 may be enforced by promoting the lipidation or the nuclear translocation of VPAC1. Moreover, these novel findings also brings research hints for the other class B GPCRs, such as PAC1 and VPAC2, which have similar free cysteine residues in the N-terminal extracellular domain.

Of course there are some important questions needed to be settled down including when the Cys37 is palmitoylation and how the palmitoylated VPAC1 is transported to the nuclear. It was reported 5-Hydroxytryptamine 4(a) receptor expressed in Sf9 cells is palmitoylated in an agonist-dependent manner [28]. Whether the palmitoylation of Cys37 in VPAC1 depends on the receptor's activation by its agonist needs the subsequent study. Furthermore, we should find out which kind of lipid rafts or which kind of membrane proteins that mediated the nuclear translocation of VPAC1. It was reported that palmitoylation aid or enhance caveolar localization of proteins and the reversibility of palmitoylation help regulate the movement of molecules into and out of caveolae in response to stimulation by agonist [29]. And palmitoylation was also reported to help some proteins into some kind of endosome [30], which delivered surface proteins to the nucleus [31]. The mechanism of the nuclear translocation of VPAC1 needs further research.

## MATERIALS AND METHODS

### Materials

All materials for cell culture and transfection reagents were from Invitrogen (Carlsbad, CA). Reagents for molecular biological techniques were obtained from Takara (Dalian, China) and Qiagen (Valencia,CA). The peptide VIP was synthesized by Qiangrao Biological Company (Shanghai, China). cDNA encoding mouse VPAC1 was from GeneCopoeia Company (Guangzhou, China). The eukaryotic expression vectors pEYFP-N (containing the gene encoding EYFP) was purchased from Yingrun Biological Company (Changsha, China).

### Construction of recombinant vector

The VPAC1-C37/A mutation was constructed by overlapping PCR using primers were designed and synthesized following Table 1 (Figure 1B). In detail, the gene encoding VPAC1-C37/A was obtained by four rounds of overlapping PCRs using forward primers F-F4, F-F3, F-F2, and F-F1 in order respectively combined with backward primer B-F5, while the gene encoding wild type VPAC1 was obtained with primer F-F1 and backward primer B-F5. Both two genes were fused into the eukaryotic expression vector pEYFP-N by double digestions with *EcoR* I/*Sac* II (TaKaRa, Dalian, China) to obtain recombinant vector expressing VPAC1-EYFP and VPAC1-C37/A -EYFP respectively.

**Table 1:** Oligonucleotide primers design

Primer	Sequence(5′–3′)
F-F1	ACTGGAGCGAATTCATGCGCCCGCCAAGTCCGCTGCCCGCCCGCTGGCTA
F-F2	AGTCCGCTGCCCGCCCGCTGGCTATGCGTGCTGGCAGGCGCCCTCGCCTGGGC
F-F3	CTGGCAGGCGCCCTCGCCTGGGCCCTTGGGCCGGCGGGCGGCCAGGCGGCC
F-F4	GGGCCGGCGGGCGGCCAGGCGGCCAGGCTGCAGGAGGAGGCGGACTATGTGCAGATGATC
B-F5	ATCGGATCCCGCGGGACCAGGGAGACTTCGGCTTGGAAGCTGGA

The underlined indicates the restriction enzyme sites of *EcoR* I/*Sac* II, the shadow indicates the mutation site coding C37/A.

### Transfection and cells expression

The CHO cells at logarithmic phase were digested and were seeded into six-well plates with a density of 2 × 10^5^cells/ml. After the cells were anchored, the recombinant vectors were transfected by Lipofectamine LTX. After 4–6 h of transfection, the culture medium was changed to DMEM containing 10% fetal bovine serum (FBS); and the CHO cell clones with stable expression VPAC1-EYFP (named VPAC1-CHO) and VPAC1-C37/A (named VPAC1-C37/A -CHO) respectively were screened using antibiotic G418 at the final density of 2.5 mg/ml for about 30 days, and the expression levels of receptors were detected by fluorescent quantification and western blotting.

### Fluorescence confocal microscopy

Cellular trafficking of VPAC1-EYFP and VPAC1-C37/A-EYFP were evaluated by visualizing EYFP fluorescence in CHO cells using appropriate spectral settings (excitation, 488 nm argon laser; emission, 545 nm filter; pinhole diameter 2.3 airy units) of the confocal microscope (LSM 510 META; Zeiss, Thornwood, NY) equipped with a Plan-Apochromat63×/1.4 numerical aperture oil objective. For the detection the effect of VIP on the trafficking of VPAC1-EYFP and VPAC1-C37/A-EYFP, the VPAC1-CHO and VPAC1-C37/A-CHO cells grown on Petri dish were washed with PBS for twice and submitted to serum-withdrawal overnight, then the cells were cultured with or without VIP (0.1 nM) for 60 min before the fluorescent confocal microscopy images were collected.

### Fluorescence quantification

For fluorescent quantification of the total receptor expression, VPAC1-CHO and VPAC1-C37/A-CHO and CHO cells at logarithmic phases were collected, counted and lysised by ultrasonication fully, and the rough lysate were submitted to the fluorescent quantification on the multifunctional fluorescence detector Victor3 1420 (PE, USA) with exciting/emission light wavelengths of 460 ± 30 nm/535 ± 30 nm. And the expression of EYFP-tagged receptor was quantified using the formula: Unit cell fluorescence value = the light densities/ cells counts. The experiments were performed in parallel with at least five replicates and were repeated three times. For fluorescent quantification of the receptor transporting into the nuclear, the cell lysates of VPAC1-CHO and VPAC1-C37/A-CHO cells were submitted to the nuclear extraction using KeyGEN Nuclear and Cytoplasmic Protein Extraction Kit (KeyGEN, Shanghai, China), and the nuclear fraction were further lysis by ultrasonication then were submitted to the fluorescence quantification and protein concentration quantification using BCA protein assay kit (KeyGEN, Shanghai, China). The intranuclear EYFP-tagged receptor was calculated using the formula: The intranuclear receptor = the fluorescence densities in nucleus /protein concentration of nucleus.

### VIP binding assay

The membrane preparation and competition binding assay were performed following the protocol described by Couvineau A. et al. [13]. Membranes (50 ug protein/ml) were incubated at equilibrium for 60 min at 30°C with 1 nM [^125^I]-VIP with or without unlabeled VIP. Specific binding was calculated between the amount of [^125^I]-VIP bound in the absence (total binding) and presence (nonspecific binding) of 2 uM unlabeled peptides. The binding data of [^125^I]-VIP to the membrane receptor was further calibrated by the EYFP fluorescence signals of the membrane representing the amount EYFP-tagged VPAC1 or VPAC1-C37/A on the membrane.

### Western blotting

For detecting the nuclear translocation of VPAC1-EYFP and VPAC1-C37/A-EYFP induced by VIP (0.1 nM), the plasma fraction without nuclear fraction and the nuclear fraction of VPAC1-CHO cells and VPAC1-C37/A-CHO cells were prepared using KeyGEN Nuclear and Cytoplasmic Protein Extraction Kit and submitted to 10% SDS-PAGE and western blotting using anti-GFP monoclonal antibody (Amyjet Scientific Inc, China), which recognized both GFP and EYFP. Western blotting of β-actin antibody (Abclonal, USA) was used as marker for plasma protein, while nucleoporin p62 antibody (Santa Cruze, USA) as maker for nuclear fraction.

### Proliferative activity assay

For detecting the proliferative activity induced by VIP, VPAC1-CHO, VPAC1-C37/A-CHO and CHO cells were seeded with similar density of 2 × 10^5^ cells/ml, and the washed cells were submitted to culture with 0.1 nM-1000 nM VIP in DMEM for 24 h. The cell viability was determined by MTT assay, while the treatment without VIP was used as control. For growth curve assay, VPAC1-CHO, VPAC1-C37/A-CHO and CHO cells were seeded with similar density of 2 × 10^4^ cells/ml and cultured with DMEM containing 0.1 nM VIP and 1% FBS. And the cell viabilities were determined using the colorimetric MTT assay (Methylthiazolete trazolium bromide, Sigma, USA) after the culture for 1 d, 2 d, 3 d and 4 d. Six parallel wells were set for each test, and each test was repeated three times.

### CPT induced apoptosis

Cultured VPAC1-CHO, VPAC1-C37/A-CHO and CHO cells with 80% confluence were washed with PBS for twice and serum-withdrawal overnight. After the cells were cultured with DMEM containing 0.1nM VIP for 60 min with or without palmitoylation inhibitor 2-bromopalmitate (2-BP) (50 uM) respectively, the cells were then subjected to culture with CPT (50 uM) for 12 h. The cells treated without inhibitors or CPT was used as normal control. The remaining live cells were assayed using the colorimetric MTT assay. The remaining cell viability was calculated using the formula: Remaining cell viability (%) = MTT value with CPT/MTT value without CPT × 100%. In addition, the intracellular level of the anti-apoptosis factor Bcl-2 was determined using Bcl-2 Elisa Kit (Beyotime, Shanghai, China). And the data were all normalized with the plasma protein concentrations. Furthermore, the TUNEL (Terminal transferase dUTP nick end labeling) assay and flow cytometry combined dyeing with Annexin V/PI were also used to assay the apoptosis.

### Bioinformatics prediction of the modification on Cys37 of VPAC1

The sequence of VPAC1 extracellular domain 1-142 aa (with signal peptide) and mature VPAC1 with 31-457 aa (without signal peptide) were submitted to the prediction of the potential modification on cysteines using the online service software, including lipid modification using GPS-Lipid (http://lipid.biocuckoo.org/webserver.php), S-nitrosylation using GPS-SNO (http://sno.biocuckoo.org/online.php), plamitoylation using CSS-Palm (http://nbapalm.biocuckoo.org/), sumoylation using GPS-SUMO (http://sumosp.biocuckoo.org/online.php), and phosphorylation using GPS (http://gps.biocuckoo.org/) for qualitative and quantitative analysis.

### Acyl-biotin exchange assay

The acyl-biotin exchange assay was conducted imitating the method described by Zhang Y, et al. [14]. In brief, the VPAC1-CHO and VPAC1-Cys37/Ala-CHO cell lysates were incubated overnight with 1M NEM (Sigma, USA) at 4°C to block all the free sulfhydryls. Fresh 1M HA (Sigma, USA) was added and the culture lasted for 1 h at room temperature to cut off the thioester bond of palmitoylation to produce extra free sulfhydryls. And 4M biotin-HPDP (ProteoChem, USA) was added later to react with the free sulfhydryls produced by HA to achieve the biotin exchange labeling of the palmitoylation. Then streptavidin-agarose mini-column (Sigma, USA) were used to gather the biotin-labeling proteins, and the final elution from the streptavidin-agarose mini-column was submitted to 10% SDS-PAGE and western blotting using monoclonal antibody recognizing EYFP (Amyjet Scientific, China). The treatment without HA was used as systemic control.

### Click chemistry palmitoylation assay

Click chemistry palmitoylation assay was conducted imitating the method described by Coleman DT, et al. [15]. In brief, after the metabolic labeling the palmitoylated proteins with palmitate orthologs alkyne-linked 17-Odya (Cayman Chemical, USA), the cells lysates were further processed through the click reaction between biotin-azide (Cayman Chemical, USA) and alkyne-linked 17-Odya, which make the palmitoylated proteins covalently cross-linked with biotin at the S-palmitoylation sites. The biotinylated protein were pulled down from the cells lysates using streptavidin-agarose mini-column (Sigma, USA), and then were subjected to western blotting using monoclonal antibody recognizing EYFP (Amyjet Scientific, China).

### Statistical Analysis

Statistical analysis was performed with GraphPad Prism, using the unpaired t test. Differences with *p* < 0.05 were considered to be statistically significant. All results were expressed as means ± SEM (standard error of the mean).

## Abbreviations

VPAC1: Vasoactive intestinal peptide receptor 1, also recognized by pituitary adenylate cyclase activating polypeptide (PACAP)
VIP: Vasoactive intestinal peptide
EYFP: Enhanced yellow fluorescent protein
CPT: camptothecin
2-BP: Palmitoylation inhibitor 2-bromopalmitate
HA: hydroxylamine
NEM: N-ethylmaleimide
